# Generation and characterization of temperature-sensitive alleles of the glucanosyltransferase Gas1 in Schizosaccharomyces pombe

**DOI:** 10.17912/micropub.biology.001144

**Published:** 2024-03-05

**Authors:** Isaac V. Howard, Bita Tavafoghi, Maya G. Igarashi, Liping Ren, Alaina H. Willet, Kathleen L. Gould

**Affiliations:** 1 Department of Cell and Developmental Biology, Vanderbilt University School of Medicine, Nashville, TN, US; 2 Biophysical Sciences, Current address: University of Chicago, Chicago, IL, US

## Abstract

The *Schizosaccharomyces pombe* Gas family of β-1,3-glucanosyltransferases modify the cell wall by elongating β-1,3-glucan chains. While *gas1Δ* cells are inviable under standard laboratory growth conditions, they are viable in the presence of an osmotic stabilizer. Even under these conditions however, *gas1Δ* cells are slow-growing and display cell separation and morphology defects. Here, we isolated and characterized two *gas1* temperature-sensitive alleles. Our data support that Gas1 is the primary *S. pombe* β-1,3-glucanosyltransferase important for cell separation and cell viability and provide useful tools for further analysis of *S. pombe* cell wall formation.

**Figure 1. Characterization of gas1 mutant alleles.**
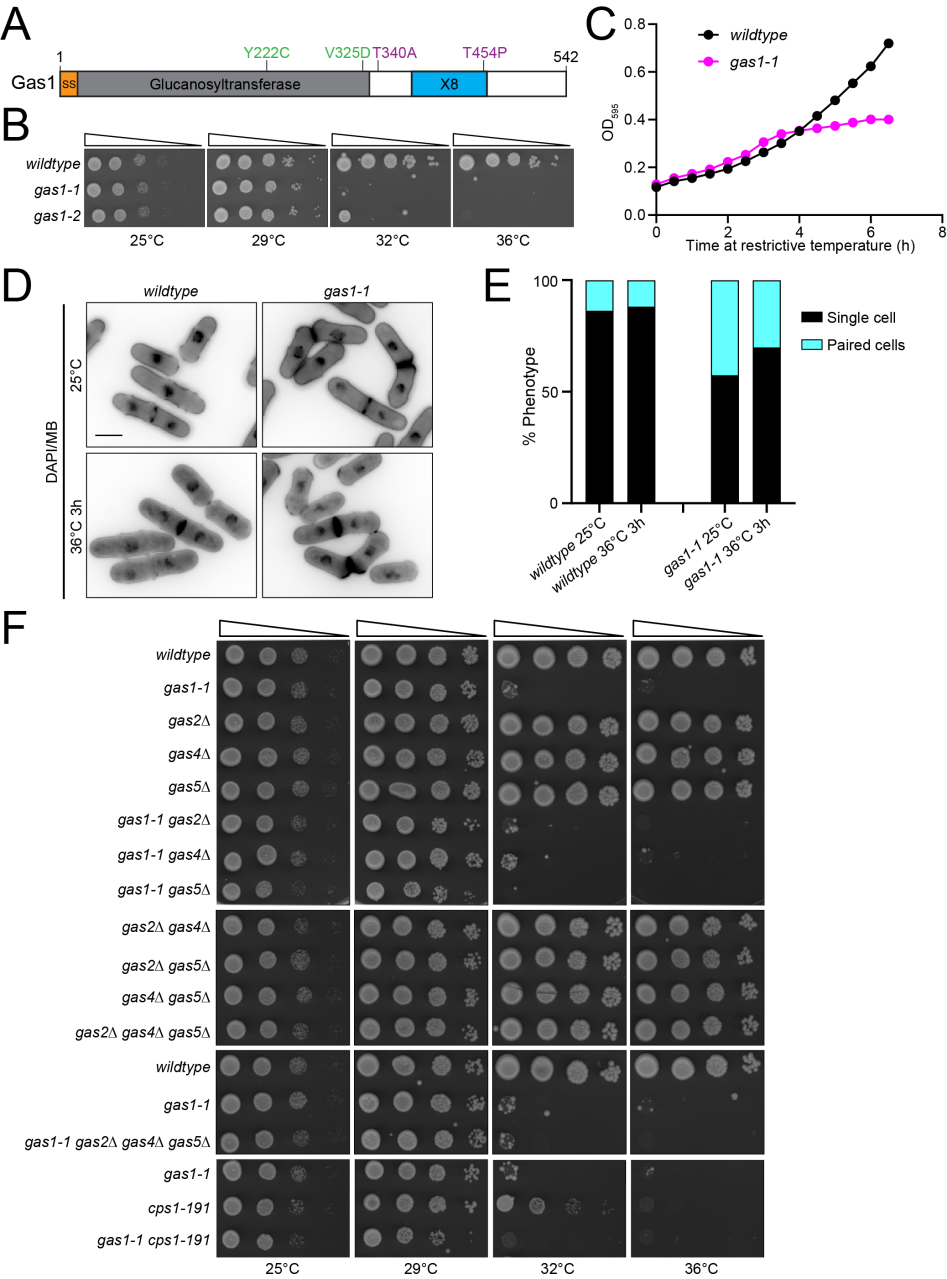
(A) A schematic, drawn to scale, of the protein encoded by *gas1* . The amino acid substitutions in *gas1-1* are indicated in green and those in *gas1-2* are indicated in purple. Signal sequence (SS). (B, F) The indicated strains were grown in liquid YE at 25˚C until they reached mid-log phase. Then, 10-fold serial dilutions were made and 2.5 µL of each was spotted on YE agar plates and incubated at the indicated temperatures for 3 days prior to imaging. (C) Growth curve of wildtype and *gas1-1* cells following a temperature shift from 25°C to 36°C. OD595 was measured every 30 minutes for 6.5 hours. (D) Wildtype and *gas1-1* cells were grown at 25°C and then shifted to 36°C for 3 hours prior to fixation with 70% ethanol. Samples were taken at each timepoint. Cells were washed three times with PBS and then stained with methyl blue (MB) and DAPI prior to imaging. (E) The indicated phenotypes were quantified from the same experiment as in D. n ≥ 490 for each.

## Description

The cell wall is important for proper cell shape and protection from the environment for many organisms. How the cell wall maintains its barrier function while simultaneously being remodeled to allow for cell growth and division is not fully understood (Cosgrove, 2005; Free, 2013; Silhavy et al., 2010) . The fission yeast *Schizosaccharomyces pombe* contains a cell wall primarily composed of ɑ1,3-glucans, β1,3-glucans, β1,6-glucans and galactomannan (Bush et al., 1974; Humbel et al., 2001; Osumi et al., 1998) . The glucans are synthesized by the essential glucan synthases Bgs1 , Bgs3 , Bgs4 and Ags1 (Cortés et al., 2005; Liu et al., 1999; Martín et al., 2003; Vos et al., 2007). The *S. pombe* genome encodes many proteins that modify the cell wall including the β-1,3-glucanosyltransferase Gas family of proteins (Harris et al., 2022) . These proteins belong to the glycoside hydrolase 72 (GH72) family of enzymes (Henrissat & Davies, 1997) and are glycosyl-phosphatidylinositol (GPI)-anchored at the cell surface (de Groot et al., 2007) . *S. pombe* contain 4 of these enzymes; Gas1 , Gas2 , Gas4 and Gas5 (Harris et al., 2022) . They function to elongate β-1,3-glucan chains by first internally cleaving a β-1,3-glucan chain and then appending the newly generated reducing end to a non-reducing end of an acceptor β-1,3-glucan chain (de Medina-Redondo et al., 2010; Mouyna et al., 2000) . Gas1 is of particular interest because deletion of the gene renders cells viable only in the presence of an osmotic stabilizer and the cells have severe defects in growth and morphology (de Medina-Redondo et al., 2010; Hayles et al., 2013; Kim et al., 2010) . Consistent with their biochemical activity, the cell wall of *gas1Δ* cells had shorter β-1,3-glucan chains compared to that of wildtype cells (de Medina-Redondo et al., 2010) . However, the cellular function of Gas1 has not been assessed under standard (non-osmotic stabilizing) conditions.

Here, we isolated two new temperature-sensitive *gas1* alleles obtained by an error-prone PCR method (Tang et al., 2019) . The two alleles were sequenced; *gas1-1* contains Y222C and V325D substitutions within the glucanosyltransferase domain and *gas1-2* contains T340A and T454P substitutions downstream of the catalytic domain ( Figure 1A ). We next determined the range of temperature-sensitivity for each *gas1* mutant by spotting the cells at a variety of temperatures ( Figure 1B ). *gas1-1* grew similarly to wildtype at 25°C and 29°C but showed almost no growth at 32°C or 36°C. *gas1-2* grew like wildtype at 25°C and 29°C, had reduced growth at 32°C, and no growth at 36°C ( Figure 1B ). Further characterization of the more severe *gas1-1* mutant was done in liquid media. Wildtype and *gas1-1* cells were grown at 25°C and then shifted to 36°C ( Figure 1C ). This analysis revealed the *gas1-1* cells almost completely ceased growth after 4 hours at 36°C while the wildtype cells continued to grow exponentially ( Figure 1C ). When the cells were fixed and stained with DAPI and methyl blue (MB) to visualize DNA and the cell wall, respectively, we found that a much larger portion of *gas1-1* cells were paired at 25°C and 36°C compared to wildtype ( Figure 1D -E). These results indicate that *gas1-1* cells have a cell separation defect, similar to what was observed for *gas1Δ* cells in sorbitol containing media (de Medina-Redondo et al., 2010) .

To determine if any other *S. pombe* Gas proteins have a similar function to Gas1 , we performed a genetic analysis. While *gas1Δ* is lethal under standard conditions, the gene deletions of *gas2* , *gas4* or *gas5* are viable (de Medina-Redondo et al., 2010; Hayles et al., 2013; Kim et al., 2010) . We therefore combined *gas1 -1* with each additional single deletion mutant and assayed cell growth. No further growth defect was observed when *gas1-1* was combined with *gas2Δ* or *gas4Δ* but there was a slight negative genetic interaction with *gas5Δ* ( Figure 1F ). Further, the triple deletion mutant, *gas2Δ gas4Δ gas5Δ,* did not result in any growth defect compared to wildtype cells and the quadruple mutant, *gas2Δ gas4Δ gas5Δ gas1-1* , grew similarly to *gas1-1* ( Figure 1F ). We conclude that the Gas proteins do not appear to have redundant functions. This result is expected for *gas4* which is only expressed in meiosis and *gas2* which has a different cell-cycle dependent expression pattern from *gas1* (de Medina-Redondo et al., 2008, 2010) . *gas1* and *gas5* have similar expression patterns but biochemical experiments suggest they may have different substrate preferences related to glucan chain length, which may also explain why *gas5Δ* does not show a strong negative genetic interaction with *gas1-* 1 (de Medina-Redondo et al., 2010) .

Lastly, we predicted that *gas1-1* cells combined with other alleles that affect cell wall function would lead to synthetic growth defects. To assess this, we combined *gas1-1* with *cps1-191* which contains a mutation in the gene that encodes the β-1,3-glucan synthase Bgs1 (Liu et al., 1999) . Indeed, the double mutant *gas1-1* *cps1-191* grew less well compared to each single mutant ( Figure 1F ), suggesting Gas1 does indeed play an important role in promoting proper cell wall assembly.

In conclusion, *gas1* is important for cell separation and cell growth while *gas2* , *gas4 ,* and *gas5* do not appear to play major roles in these processes during vegetative growth. Because Gas1 has predicted 1,3-β-glucanosyltransferase activity, it is expected to work downstream of β-1,3-glucan synthases to elongate β-1,3-glucan chains. The ability to modify the cell wall in this manner may be important for structural flexibility, especially during large shape changes that are required during the cell division process.

## Methods

Yeast methods

*S. pombe* strains were grown in yeast extract (YE) and standard *S. pombe* mating, sporulation, and tetrad dissection techniques were used to construct new strains (Moreno et al., 1991) .

Molecular biology methods

*gas1* alleles were sequenced by generating a PCR product with an oligonucleotide 100 bp upstream of the start site (GTCATTTTTTGACATTTTCATTCTTGC) and a reverse oligonucleotide within *kanMX6* (Integrated DNA technologies). The PCR product was sequenced with oligonucleotides at 50 bp upstream of the *gas1* start site (CATAACTTTTTATCTCTTTTAATACCCTG) and 600 bp within the *gas1* coding sequence (CGAAGAGGTTACTCGTGACCCTATGG). The *gas4* gene deletion was made as previously described (Chen et al., 2015) .

Isolation of temperature sensitive alleles with error-prone PCR

Temperature-sensitive mutants of *gas1* were constructed and isolated based on the previously described protocol (Tang et al., 2019) but using EX taq polymerase (Takara, 4025) and accompanying dNTPs (Takara, RR01BM).

Microscopy and image analysis

Strains for fixed-cell imaging experiments were grown at 25°C in YE and then shifted to 36°C for 3 hours. Cells were fixed with 70% ethanol for DAPI and methyl blue (MB) staining as described previously (Roberts-Galbraith et al., 2009) . Images were acquired using a Zeiss Axio Observer inverted epifluorescence microscope with Zeiss 63× oil (1.46 NA) and captured using Zeiss ZEN 3.0 (Blue edition) software. A singular medial Z slice was obtained. All images were further processed using ImageJ (Schindelin et al., 2012) . Graphs were constructed with Prism 8.0 (GraphPad Software).

## Reagents

The strains used in this study and their genotypes are listed below.

**Strain** **Genotype** **Source**

KGY246 *ade6-M210 leu1-32 ura4-D18 h ^-^* Lab stock

KGY5486-2 *gas1-1(Y222C, V325D):kanMX6 ade6-M210* This study

*leu1-32 ura4-D18 h ^-^*

KGY1044 *gas1-2(T340A, T545P):kanMX6 ade6-M210* This study

*leu1-32 ura4-D18 h ^-^*

KGY16388 *gas2Δ::kanMX6* *ade6-M210 leu1-32 ura4-D18* *h ^-^* Lab stock

KGY5010-2 *gas4Δ::kanMX6* *ade6-M210 leu1-32 ura4-D18* *h ^-^* This study

KGY954-2 *gas5Δ::kanMX6* *ade6-M210 leu1-32 ura4-D18* *h ^+^* Bioneer, v3

KGY3811-2 *gas1-1:kanMX6* *gas2Δ::kanMX6* *ade6-M210* This study

*leu1-32 ura4-D18* *h ^+^*

KGY5732-2 *gas1 -1:kanMX6* *gas4 Δ::kanMX6* *ade6-M210* This study

*leu1-32 ura4-D18* *h ^+^*

KGY3812-2 *gas1 -1:kanMX6* *gas5 Δ::kanMX6* *ade6-M210* This study

*leu1-32 ura4-D18* *h ^+^*

KGY5896-2 *gas2 Δ::kanMX6* *gas4 Δ::kanMX6* *ade6-M210* This study

*leu1-32 ura4-D18* *h ^+^*

KGY3814-2 *gas2 Δ::kanMX6* *gas5 Δ::kanMX6* *ade6-M210* This study

*leu1-32 ura4-D18* *h ^+^*

KGY5897-2 *gas4 Δ::kanMX6* *gas5 Δ::kanMX6* *ade6-M210* This study

*leu1-32 ura4-D18* *h ^+^*

KGY6100-2 *gas2 Δ::kanMX6* *gas4 Δ::kanMX6* *gas5 Δ::kanMX6* This study

*ade6-M210 leu1-32 ura4-D18* *h ^+^*

KGY5902-2 *gas1 -1:kanMX6* *gas2 Δ::kanMX6 gas4 Δ::kanMX6* This study

*ade6-M210 leu1-32 ura4-D18* *h ^+^*

KGY5900-2 *gas1 -1:kanMX6* *gas2 Δ::kanMX6* *gas5 Δ::kanMX6* This study

*ade6-M210 leu1-32 ura4-D18* *h ^+^*

KGY5899-2 *gas1 -1:kanMX6* *gas4 Δ::kanMX6* *gas5 Δ::kanMX6* This study

*ade6-M210 leu1-32 ura4-D18* *h ^+^*

KGY6101-2 *gas1 -1:kanMX6* *gas2 Δ::kanMX6 gas4 Δ::kanMX6* This study

*gas5 Δ::kanMX6* *ade6-M210 leu1-32 ura4-D18* *h ^+^*

KGY17141 *cps1 -191* *ade6-M210 leu1-32 ura4-D18* *h ^+^* Lab stock

KGY6098-2 *gas1 -1:kanMX6* *cps1 -191* *ade6-M210 leu1-32 ura4-D18* *h ^+^* This study
